# Emerging Role for Antioxidant Therapy in Protection Against Diabetic Cardiac Complications: Experimental and Clinical Evidence for Utilization of Classic and New Antioxidants

**DOI:** 10.2174/157340308786349453

**Published:** 2008-11

**Authors:** Michael F. Hill

**Affiliations:** From the Department of Medicine, Division of Cardiovascular Medicine, Vanderbilt University Medical Center, Nashville, TN 37232, USA

**Keywords:** Antioxidants, diabetic cardiomyopathy, diabetes mellitus, heart failure, myocardial infarction, oxidative stress.

## Abstract

Diabetes mellitus (DM) markedly potentiates the risk of cardiovascular morbidity and mortality among individuals with diabetes as compared to the non-diabetic population. After myocardial infarction (MI), DM patients have a higher incidence of death than do non-diabetics. The excess mortality and poor prognosis of these patients results primarily from the development of recurrent MI and heart failure (HF). Although several lines of evidence support a role for increased oxidative stress in a range of cardiovascular diseases, clinical trials examining the therapeutic efficacy of antioxidants have yielded conflicting results. The reasons for these incongruous results is multifactorial. An underlying theme has been lack of patient inclusion based on elevated indices of oxidative stress which could have diluted the population susceptible to benefit in the clinical trials. Laboratory evidence has accumulated indicating that oxidative stress is dramatically accentuated in cardiac abnormalities inherent in DM. In this review, we provide the emergence of experimental and  clinical evidence supporting antioxidant supplementation as a cardioprotective intervention in the setting of DM. Specifically, focus will be directed on preclinical animal studies and human clinical trials that have tested the effect of antioxidant supplements on MI and HF events in the presence of DM.

## INTRODUCTION

An estimated 77% of U.S. hospitalizations for complications of diabetes mellitus (DM) are linked to cardiovascular disease [1]. Patients with DM have substantially higher cardiovascular morbidity and mortality than their non-diabetic counterparts. According to a new study commissioned by the American Diabetes Association (ADA), 20.8 million children and adults in the United States have DM, with 1.5 million new cases of DM diagnosed annually (http://www.diabetes.org/diabetes-statistics/prevalence.jsp; accessed April, 2008). A recent multinational study indicated that cardiovascular disease was the most common underlying cause of death, accounting for 44% of deaths in type 1 (insulin-dependent) DM and 52% of deaths in type 2 (non-insulin-dependent) DM [2]. The reduced survival rate of patients with DM has been reported to result primarily from the more frequent development of heart failure (HF) and recurrent myocardial infarction (MI) [1, 3-9]. The combination of a specific diabetic cardiomyopathy and co-morbidities associated with DM (including obesity, dyslipidemia, hypertension, and coronary artery disease), independently and cooperatively, lead to structural and functional abnormalities of the heart culminating in cardiac dysfunction and ultimately HF [10, 11]. With the prevalence of DM rising and approaching pandemic proportions worldwide [12] due, in part, to rising rates of obesity and attendant metabolic syndrome (MS) [11], it is likely that the incidence of cardiovascular morbidity and mortality will continue to increase in this high-risk population.

Available evidence from experimental models as well as observational studies have reported that DM increases oxidative stress and that the onset of DM and its cardiac complications, including diabetic cardiomyopathy, are closely associated with accentuated oxidative stress [13-18]. However, clinical trials examining the use of antioxidants specifically in patients with DM is limited. Although randomized placebo-controlled clinical trials evaluating the efficacy of antioxidants in preventing cardiovascular events have yielded conflicting results [19-26], none of these studies used, as endpoints, biomarkers that were specific and sensitive for oxidative stress. Moreover, the ability of the antioxidants tested to reduce significantly the levels of oxidative stress in study participants in these trials was never determined. The absence of knowledge regarding the effects of antioxidants on specific markers of oxidative stress in these studies has rendered the lack of consistent cardiovascular benefit in these patients difficult to interpret and open to further investigation. In that regard, the emergence of a reliable and specific marker of oxidative stress status, i.e., measurement of F_2_-isoprostanes, has led to a burgeoning of experimental and clinical studies exploring the ability of antioxidants to mitigate cardiovascular disease in DM. The purpose of this review is to highlight accumulating evidence in favor of antioxidants to be used as prophylactic and therapeutic agents against MI and HF events in the setting of DM.

## OXIDATIVE STRESS

Oxidative stress is defined as an imbalance between the generation of free oxygen radicals (FORs) and the antioxidant defense system. In the simplest of terms, a free radical is any atom or molecule that has an unpaired electron in their outer orbit making that atom or molecule a highly reactive species. Free radical production occurs via the addition of an electron or by its removal in a reduction/oxidation reaction. Due to its unique diradical configuration, oxygen is a major intracellular source of radical species. A sequential univalent reduction of oxygen gives rise to reactive intermediate products [27, 28]. A single electron reduction of oxygen gives rise to superoxide anion (O_2_^-^), which can act as both a reducing and an oxidizing agent. The relatively short half life of superoxide anion limits its diffusion away from the site of its generation. The divalent reduction of oxygen yields the nonradical species, hydrogen peroxide (H_2_O_2_). H_2_O_2 _has a relatively long half life and therefore can travel significant distances, causing damage at sites distant from its origin. A three electron reduction of oxygen yields the hydroxyl radical (OH^-^), which is the most reactive and potent of all the free oxygen radicals. Addition of a fourth electron results in the formation of water. All of these reactive oxygen intermediates are called activated oxygen species and are collectively termed as partially reduced forms of oxygen (PRFO) [29]. Endogenous sources of free radicals are the numerous enzyme and non-enzyme systems located in the subcellular membranes, plasma membrane, and blood cell elements.

## ANTIOXIDANTS

Due to a continuous generation of PRFO during cardiac cell metabolism, a number of protective enzymatic as well as non-enzymatic antioxidants have evolved constituting an antioxidant reserve [29] that act to limit the tissue concentration of these highly reactive species. A proper balance between the generation of PRFO and the antioxidant defense system is critical for the maintenance of a normal myocardial cell structure and function.

### Enzymatic Antioxidants

Three of the most important enzymes commonly present in most cells are superoxide dismutase (SOD), catalase (CAT), and glutathione peroxidase (GSHPx). SOD catalyzes the dismutation of O_2_^-^ to H_2_O_2_. Both CAT and GSHPx catalyze the reduction of H_2_O_2_ to water, thereby preventing the formation of the potent OH^-^. A detailed account of these antioxidant enzymes has been provided in several reviews [27, 30, 31].

### Non-Enzymatic Antioxidants

The most important non-enzymatic antioxidants are vitamin E, vitamin C and glutathione. Vitamin E is an important micronutrient that also functions as an antioxidant. It acts to protect polyunsaturated fatty acids (PUFA) from oxidation by interrupting the chain of membrane lipid peroxidation and thus is also referred to as a “chain-breaking” antioxidant [32]. Since vitamin E is lipid soluble, deficiency of myocardial vitamin E levels result in the increased susceptibility of myocardial cell membranes to FOR mediated lipid peroxidation [33, 34]. Lipid peroxidation chain reaction in the membrane is “initiated” by the removal of hydrogen from the unsaturated site in a fatty acid resulting in the production of a lipid radical. The radical thus generated can interact with more PUFA and the chain is “propagated”. During this process, the chroman head group of vitamin E encounters a lipid radical, resulting in the generation of a vitamin E radical. The vitamin E radical is much more sta-ble, and less reactive, than the lipid radical. This effectively terminates lipid peroxide mediated chain reactions. The resultant vitamin E radical is incapable of any further free radical scavenging activity. Thus, it must be regenerated back to a functional vitamin E molecule. Vitamin C, present in the water phase, regenerates vitamin E to the reduced form and therefore acts to spare the functional form of vitamin E [27].

Glutathione is present in high concentrations as reduced (GSH) glutathione with minor fractions being oxidized (GSSG) glutathione [35]. GSH as a co-substrate of GSHPx, plays an important role in the removal of H_2_O_2 _as well as other organic peroxides, thus preventing the peroxidation of membrane lipids [36]. Study of changes in glutathione status provides important information on cellular oxidative events and tissue accumulation and/or release of GSSG.

## OXIDATIVE STRESS AND TISSUE INJURY

Under normal physiological conditions, the tissue concentration of FORs is limited due to the existence of a delicate balance between the generation of PRFO and the antioxidant defense system [37]. However, if this balance is disturbed in favor of more PRFO, either through an enhanced production or via a reduction in the endogenous antioxidant defense system or both, the heart is at risk for PRFO-mediated myocardial cell damage. Thus, changes in myocardial antioxidant status and oxidative stress may have profound effects on cardiac structure and function [38].

One mechanism of tissue damage induced by PRFO is via lipid peroxidation, in which there is formation of lipid peroxides within myocardial cell membranes. This process is initiated when one hydrogen is abstracted from PUFA by a free radical to form fatty acid radicals [27]. In addition, PRFO can cause oxidation of the thiol groups in proteins and can directly interact with nucleic acids. Thus, in these chemical reactions, there are structural as well as functional modifications in the macromolecules leading to modifications/alterations in cell and organ function.

## OXIDATIVE STRESS IN DM AND CARDIOVASCULAR DISEASE

### Experimental Studies

DM is characterized by a variety of metabolic abnormalities, principal among which are hypoinsulinemia (type 1 DM), insulin resistance (type 2 DM), and hyperglycemia (both type 1 and type 2 DM). Among the sequelae of hyperglycemia, increased oxidative stress has captured considerable attention as a potential pathophysiological mechanism for cardiac dysfunction and HF in DM. An increase in the production of reactive oxygen species (ROS) in DM has been shown to result from the hyperglycemia-induced enhancement in glucose auto-oxidation, protein glycation (advanced glycation endproducts [AGEs]), and subsequent oxidative degradation of glycated proteins [39-43]. Early experimental evidence implicating ROS in diabetic cardiac complications was mainly derived from reports evaluating the rate of lipid peroxidation. Increased cardiac levels of thiobarbituric acid reactive substances (TBARS) and lipid peroxides were observed in rats treated with streptozotocin (STZ), a model of type 1 DM that mimics events observed in type 1 non-atherogenic diabetic cardiomyopathy [13, 14, 44]. However, these markers of lipid peroxidation were subsequently shown to lack specificity [45, 46]. Consequently, definitive evidence for the association of oxidative stress with diabetic heart disease from these studies was lacking.

F_2_-isoprostanes, a novel class of prostaglandin F_2_-like compounds described by Roberts and Morrow [47], are formed *in vivo* by non-enzymatic free radical-catalyzed peroxidation of arachidonic acid [48, 49]. An important aspect of the discovery of isoprostanes is that measurement of F_2_-isoprostanes has emerged as one of the most reliable approaches to assess oxidative stress status *in vivo* [49]. Of these, 8-*iso*-prostaglandin F_2α_ (8-*iso* PGF_2α_) has recently been shown to be a specific and sensitive quantitative index of oxidative stress *in vivo* [50]. In that regard, we recently undertook studies examining the myocardial formation of 8-iso PGF_2α_ in type 1 DM complications involving the heart [51-53]. We found that the levels of 8-*iso* PGF_2α_ were significantly higher in the surviving myocardium of diabetic post-MI rats compared to the non-diabetic MI rats concomitant with an increased functional severity of HF [51]. In STZ rats with type 1 diabetic cardiomyopathy, left ventricular (LV) levels of 8-*iso* PGF_2α_ were significantly increased *in vivo* [52, 53] which coincided with a specific ‘type 1 diabetic’ pattern of cardiac proteome changes [52]. Collectively, these experimental animal studies demonstrate that cardiac complications of DM are associated with greater myocardial oxidative stress burden.

### Clinical Studies

Increased levels of TBARS and lipid hydroperoxides have been observed in the plasma of both types of diabetic patients [54-57]. Consistent with the concept of enhanced lipid peroxidation, increased *in vivo* formation of F_2_- isoprostanes such as 8-*iso* PGF_2_α have been documented to occur in the plasma and urine of diabetic patients [58, 59]. Elevated levels of PRFO products in diabetic patients have also been reported [60].

It has been reported that hyperglycemia increases nitric oxide (NO) and O_2_^-^ generation [61]. The reaction between NO and O_2_^- ^leads to inactivation of NO and production of the potent oxidant peroxynitrite (ONOO^-^) [62]. In that regard, a study performed on patients with acute coronary syndrome undergoing coronary bypass surgery showed that diabetic ventricular biopsy specimens contained higher levels of O_2_^-^ and nitrotyrosine during myocardial ischemia [63], indicating enhanced free radical-mediated oxidative stress damage. Moreover, there is evidence that in individuals with DM, nitrotyrosine levels are increased after MI [64], indicating elevated oxidative stress. Recently, it has been shown that both lipid hydroperoxides and protein nitrotyrosine markers of oxidative stress are increased to a greater extent in patients with DM than in those without DM who undergo coronary bypass grafting surgery for coronary artery disease [65]. Thus, these studies clearly demonstrate that cardiac complications in patients with DM are associated with a greater propensity for oxidative stress.

## CARDIOPROTECTIVE EFFECTS OF ANTIOXIDANT THERAPY ON THE DIABETIC HEART

### Evidence from Experimental Models

#### Vitamin E

Natural vitamin E is a mixture of tocopherols and tocotrienols (α-, β-, γ-, δ-tocopherol, and α-, β-, γ-, δ-tocotrienol), produced only by plants [66]. Of these, α- tocopherol has the highest antioxidant potency [66]. Vitamin E is commercially available as either a mixture of naturally occurring tocopherols and tocotrienols, synthetic α- tocopherol (which consists of the eight possible stereoisomers in equal amounts), or a mixture of the synthetic tocopherol esters [67]. Its presence in biological membranes is thought to represent the major defense system against free-radical mediated lipid peroxidation.

##### Anti-Atherogenic Effects of Vitamin E

Supplementation with vitamin E to diabetic BALB/c mice, an experimental mouse model of atherosclerosis secondary to both hypercholesterolemia and hyperglycemia, was shown to retard coronary atherosclerosis accelerated by DM [68]. The beneficial effect of vitamin E observed in this model was demonstrated to be due to a reduction in oxidative stress and not secondary to a decrease in plasma glucose or cholesterol, since their respective plasma concentrations remain unchanged in the diabetic mice supplemented with vitamin E [68]. Furthermore, it has been recently reported that macrophages from diabetic BALB/c mice are under excess oxidative stress and that the antioxidant vitamin E can attenuate macrophage oxidative stress which exists in DM and leads to accelerated atherosclerosis development [69].

##### Prophylactic Effects of Vitamin E Against HF in Diabetic Cardiomyopathy

**Fig.(1):**
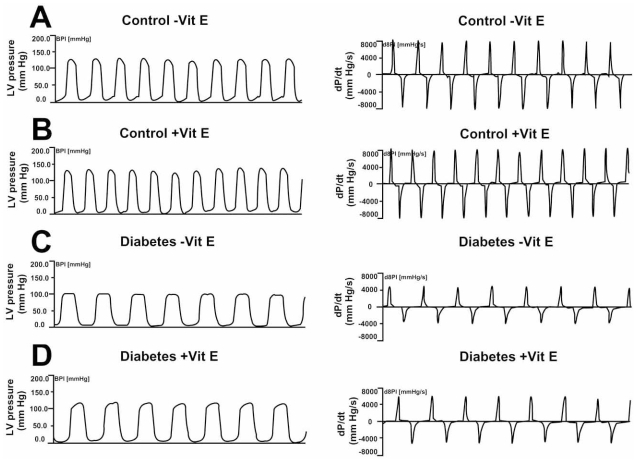
Representative left ventricular (LV) pressure recordings and maximal rate of LV pressure rise and fall (±dP/dt) obtained from control and STZ-induced diabetic rats with and without vitamin E (Vit E) treatment: (**A**) control rat maintained on a basal, un-supplemented vitamin E (-Vit E) diet; (**B**) control rat maintained on a vitamin E-supplemented (+Vit E) diet; (**C**) STZ-diabetic rat maintained on a basal, un-supplemented vitamin E (-Vit E) diet; (**D**) STZ-diabetic rat maintained on a vitamin E-supplemented (+Vit E) diet. Reprinted from Ref. 53 (Copyright 2007), with permission from Elsevier.

We recently investigated whether dietary vitamin E supplementation could confer cardioprotection against HF in type I diabetic cardiomyopathy. We found that supplementation of STZ-diabetic rats with 2000 IU of vitamin E/kg feed beginning immediately after induction of DM and continuing for 8 weeks provided significant protection against cardiac dysfunction induced by type 1 DM (Fig. **1** and Table **1**) and that this cardioprotective effect was simultaneously associated with an ability of vitamin E to blunt diabetes-induced amplification of myocardial 8-*iso* PGF_2α _and GSSG formation [53]. These findings suggest the usefulness of vitamin E supplementation during the early phases of type 1 DM for the prophylaxis of cardiomyopathy and subsequent HF.

### Metallothionein

Metallothionein (MT) is a cysteine-rich protein that has the ability to bind metals such as zinc (Zn) and copper (Cu) [70, 71]. MT also functions as a potent antioxidant owing to its high thiol content [72]. MT has been reported to protect against oxidative DNA, protein, and lipid damage induced by various ROS and reactive nitrogen species (RNS) [70, 73-75].

#### Metallothionein Prevents Diabetic Cardiomyopathy

Using a cardiac-specific, MT-overexpressing transgenic (MT-TG) mouse model, MT has been shown to prevent significantly diabetic cardiomyopathy and significantly suppress oxidative damage in the STZ-induced type 1 diabetic mouse model [76-78]. MT overexpression has also been reported to be effective in reversing the diabetic deficit in whole-heart ischemic contractility seen in OVE26 diabetic mice, a genetically predisposed type 1 diabetic mouse model of diabetic cardiomyopathy [79]. Permanent antioxidant treatment by MT overexpression specifically in cardiomyocytes has been demonstrated to be remarkably effective in ameliorating functional deficits in OVE26 diabetic cardiomyocytes by inhibiting chronic ROS production [80], indicating that ROS is essential to the pathogenesis of diabetic cardiomyopathy.

Zinc, the primary metal that binds to MT under physiological conditions, is a potent inducer of MT [81, 82]. A recent study examined whether Zn supplementation could protect against diabetic cardiomyopathy through cardiac MT induction [83]. The results from this study demonstrated that Zn supplementation to STZ-induced diabetic mice significantly prevented the increases in cardiac morphological impairment, fibrosis, and dysfunction observed in diabetic mice without Zn supplementation [83]. Zn supplementation to STZ-diabetic mice also induced a significant increase in cardiac MT expression [83]. When MT expression was silenced with the use of MT small-interfering RNA in cultured myocytes, the preventive effect of pretreatment with Zn was abolished [83]. These results provide evidence that the prevention of diabetic cardiomyopathy by Zn supplementation is predominantly mediated by an increase in cardiac MT [83] and suggest Zn supplementation, with cardiac MT induction, as a potential therapeutic approach to prevent diabetic cardiomyopathy.

### Resveratrol

Resveratrol, a polyphenolic phytoalexin compound, has been designated the principal effector constituent present in red wine [84]. Interest in this compound stems from reports that resveratrol exerts both vascular and cardioprotective effects in the non-diabetic setting [85, 86]. This beneficial effect has been attributed to the antioxidant activity of resveratrol. However, until recently, little or no data was available about the ability of the antioxidant resveratrol to protect the diabetic myocardium. In that regard, its therapeutic effects have now begun to be described in diabetes-induced cardiovascular complications.

#### Resveratrol Alleviates Diabetic-Induced Post-Ischemic Cardiac Dysfunction

Das and colleagues have demonstrated a beneficial effect of resveratrol on diabetic ischemic reperfused myocardium using two different animal models of diabetes [87, 88]. Daily oral administration of resveratrol (2.5 mg/kg body wt/day) to STZ-induced diabetic rats for 15 consecutive days after 15 days of STZ injection resulted in improved LV function following *ex-vivo* ischemia-reperfusion [87]. Cardioprotection from ischemic injury in resveratrol-treated diabetic rats was demonstrated to occur via enhanced antioxidant activity and reduced blood glucose levels [87]. STZ-diabetic rats treated with resveratrol showed a marked increase in manganese (Mn) SOD activity after ischemia-reperfusion compared to the untreated STZ-diabetic rats [87]. Since it is well documented that hyperglycemia increases the production of ROS [39-43], the authors suggest that resveratrol-induced decrease of blood glucose in conjunction with increased MnSOD activity in diabetic rat myocardium may protect the diabetic heart from oxidative stress-induced cardiac dysfunction, even in the absence of ischemia/reperfusion [87].

Das and colleagues have also shown that pre-treatment with the antioxidant resveratrol improves the recovery of post-ischemic cardiac function in isolated hearts from Zucker obese diabetic rats [88], a model of dietary-induced insulin resistance that encompasses type 2 DM. The recovery of post-ischemic cardiac function was associated with a reduction in the incidence of reperfusion-induced ventricular fibrillation (VF) [88]. These results suggest that antioxidant treatment with resveratrol can improve post-ischemic cardiac dysfunction in the insulin-resistant diabetic state.

### Glutathione Peroxidase Synthetic Mimic BXT-51072

BXT-51072 is an orally bioavailable, catalytic mimic of GSHPx (Synvista Therapeutics, Inc.) [89, 90]. GSHPx, an important defense mechanism against not only myocardial ischemia/reperfusion injury [91] but also post-MI cardiac failure [92], has been reported to be markedly decreased in diabetic coronary heart disease patients [93].

#### Infarct-Sparing Effects of BXT-Supplementation

Levy and colleagues have shown that oral administration of the antioxidant BXT-51072 at a dose of 5 mg/kg to DM mice homozygous for the haptoglobin 2 allele (Hp 2), which confers markedly less antioxidant protection against hemoglobin-induced oxidation compared with the Hp 1 allele [94], reduces MI size after myocardial ischemia-reperfusion by more than 80% [95]. In addition, non-BXT-supplemented DM Hp 2 mice exhibited significantly greater amounts of myocardial lipid peroxidation products of arachidonic acid in association with increased MI size after ischemia-reperfusion [95]. These results suggest that decreasing oxidative stress with a GSHPx antioxidant mimic may prevent increased ischemia-reperfusion injury in the setting of DM.

### Tempol

Tempol is a membrane-permeable SOD mimetic that has been shown to attenuate the effects of peroxynitrite and ROS [96]. In DM, tempol has been shown to attenuate vascular complications [97, 98]. Until recently, the protective actions of this antioxidant against myocardial complications of diabetes remained unknown.

#### Tempol Inhibits Cardiac Hypertrophy in the Pre-Diabetic Insulin-Resistant Heart

Insulin resistance is a characteristic feature of type 2 DM. The pre-diabetic insulin resistant heart exhibits many of the features of diabetic cardiomyopathy, including cardiac hypertrophy [99, 100]. *In vivo* administration of the antioxidant tempol (1 mmol/l in drinking water) for a period of 4-weeks to mice rendered insulin-resistant by deficiency of the insulin-sensitive GLUT4 transporter significantly and potently attenuated cardiac hypertrophy in concert with tempol up-regulated ventricular expression of thioredoxin-2 (confirming an antioxidant action) [101]. Further studies are ongoing in an effort to determine to what extent functional benefit is conferred by this antioxidant intervention in the setting of insulin resistance.

## EVIDENCE FROM CLINICAL TRIALS

### Vitamin E

#### Vitamin E Supplementation Reduces MI

The Secondary Prevention with Antioxidants of Cardiovascular Disease in End Stage Renal Disease (SPACE) trial [19] investigated the effect of high-dose vitamin E supplementation on cardiovascular disease outcomes in hemodialysis patients with pre-existing cardiovascular disease. In this trial, 196 hemodialysis patients with pre-existing cardiovascular disease were randomized to receive 800 IU/day vitamin E (n=97) or matching placebo (n=99) for 2 years. 42% of the patients in the placebo group had diabetes while 43% of the patients in the vitamin E group had diabetes. The primary endpoint was a composite variable consisting of: MI (fatal and non-fatal), ischemic stroke, peripheral vascular disease, and unstable angina [19]. A 46% reduction in composite cardiovascular disease endpoints in the hemodialysis patients treated with high-dose vitamin E during the 2 years’ follow-up was observed and this was largely due to a 70% reduction in total MI [19]. In that regard, evaluation of serum antioxidants in participants identified from the Pittsburgh Epidemiology of Diabetes Complications Study (EDC) cohort, a 10-year prospective follow-up study of childhood–onset (<17 years of age) type I DM patients, revealed that high serum α-tocopherol levels among type 1 DM patients with overt nephropathy were associated with a significantly lower coronary artery disease (CAD) incidence (as determined by EDC physician-diagnosed angina or MI confirmed by Q-waves on electrocardiogram) during the follow-up period in type I diabetes [102]. This cardioprotective effect of α-tocopherol among individuals with renal disease in type I DM was independent of traditional cardiovascular disease risk factors, or other serum antioxidants [102].

**Fig.(2):**
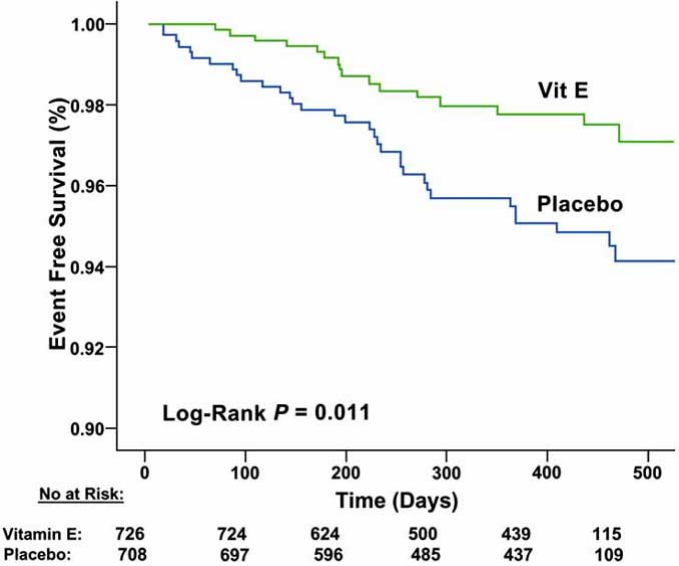
Kaplan-Meier plot of the composite end point in Hp 2-2 DM individuals allocated to vitamin E or placebo. Events are CV death, myocardial infarction, or stroke. There were 726 Hp 2-2 individuals allocated to vitamin E and 708 Hp 2-2 individuals allocated to placebo. As a reflection of the 18-month window during which participants entered the study (time 0 being the day of Hp typing) and the early termination of the study not all participants were in the study for the same duration. This is reflected in the abscissa where the number of individuals in the study (the number at risk) for a given duration is provided. There were a total of 16 patients (2.2%) who had events in the vitamin E group and 33 patients who had events in the placebo group (4.7%). There was a significant decrease in the composite end point in the vitamin E group compared with the placebo group (HR 0.47 [95% CI 0.27 to 0.82], P=0.01 by log-rank). Reprinted from Ref. 104 (Copyright 2008), with permission from the American Heart Association, Inc. and Lippincott Williams & Wilkins.

The marked increase in lipid peroxidation products of arachidonic acid in the myocardium of DM Hp 2 mice occurring after myocardial ischemia-reperfusion [95] prompted Levy and colleagues to investigate whether antioxidant therapy with vitamin E might have reduced cardiovascular events in Hp 2-2 DM patients that participated in the HOPE (Heart Outcomes Prevention Evaluation) trial (23). Hp genotype was assessed in stored blood samples from HOPE. Analysis of this HOPE cohort showed that vitamin E treatment significantly reduced MI and cardiovascular death by 43% and 55%, respectively, in these Hp 2-2 DM individuals [103]. However, due to the retrospective nature of this analysis, Levy and colleagues sought to test the validity of these findings in Hp 2-2 DM patients in a prospective, double-blind, placebo controlled clinical trial of vitamin E [104]. 1434 DM patients with the Hp 2-2 genotype were randomized to vitamin E (400 U/day) or placebo [104]. At the first interim analysis, 18 months after initiating the study, cardiovascular events were found to be significantly reduced in the cohort of Hp 2-2 DM patients receiving vitamin E compared with placebo (Fig. **2**), which led to an early termination of the study [104]. The reduction in cardiovascular events in the vitamin E-treated group of patients was in large part attributable to a significant reduction in the incidence of non-fatal MI [104]. These results suggest that this subgroup of DM patients, in particular, may derive cardiovascular benefit from treatment with the antioxidant vitamin E and in addition, provide further affirmation of the ability of vitamin E to reduce MI in the diabetic population.

The observed benefit with vitamin E in the above mentioned ongoing clinical studies are incongruent with the results from the most recent randomized placebo-controlled clinical trials [22-26]. In fact, in these trials it was very clear that vitamin E supplementation had little impact on the development of atherosclerosis in all comers, including diabetics. However, in the majority of these studies, the participants were older individuals (> 55 years of age) with advanced disease. To that end, study patients that participated in the HOPE trial [23] had preexisting coronary or peripheral artery disease, stroke, or DM in the presence of at least one additional cardiovascular disease risk factor, and the mean age was 66 years. In the GISSI-Prevenzione trial [22], study participants were exclusively patients who had survived MI and included older patients (30% of the patients randomly allocated to receive vitamin E treatment were in the 51-60 years of age category while 33% of the patients randomly allocated to receive vitamin E treatment were in the 61-70 years of age category). As a result, it is possible that the study populations in these most recent randomized placebo-controlled clinical trials represented patients in whom the coronary artery lesions were too far advanced to be amenable to antioxidant intervention with vitamin E.

### Resveratrol (Red Wine)

#### Moderate Red Wine Intake Improves Cardiac Function After MI

The preclinical observations that resveratrol treatment can improve post-ischemic cardiac dysfunction in the setting of DM [87, 88] by favorably altering oxidative stress [87] have also been demonstrated in diabetic MI patients. Marfella *et al*. examined the effects of moderate red wine intake on oxidative stress and echocardiographic parameters of functional cardiac outcome in DM patients after a first uncomplicated MI [105]. One hundred and fifteen subjects with DM who had sustained a first and recent (< 2 months) non-fatal MI were randomized to receive either one 118 ml (4-oz) glass of wine each day or no red wine (control group) for a period of 1 year [105]. After the 1 year intervention period, circulating levels of nitrotyrosine were significantly reduced in the diabetic MI patients receiving red wine compared to the diabetic MI patients that did not intake red wine [105]. In addition, moderate red wine intake among the diabetic MI subjects resulted in a significantly higher ejection fraction and significant improvement of dys-synchrony between right and left ventricular contraction and relaxation as compared to the diabetic MI patients who did not receive red wine [105]. These clinical data suggest that red wine consumption, in moderate amounts, may be an effective antioxidant therapy for preventing oxidant-dependent complications of MI in DM patients [106].

## FUTURE DIRECTIONS

Efforts are underway to achieve successful delivery of antioxidants specifically in the diabetic heart in order to maximize the potential roles of antioxidants in prophylaxis and therapy of cardiac complications of DM. The experimental results by Epstein and colleagues demonstrating that cardiac-overexpression of antioxidant transgenes permanently and specifically in hearts of type 1 and type 2 diabetic mice can prevent and reverse diabetes-induced defects in cardiomyocytes attributable to ROS production [80, 107, 108] suggests that gene therapy of antioxidants may evolve into potential approaches for the treatment of diabetic heart diseases related to oxidative stress. However, its clinical application must await the advent of effective and safe gene delivery methods.

## CONCLUSIONS

While both the American Heart Association and the American Diabetes Association remain jointly committed to preventing DM [109, 110], the explosive gain in the prevalence of obesity in adolescents over the past three decades increases the likelihood that the number of people with diagnosed DM will continue to soar in the near term. Because cardiovascular disease is the major cause of morbidity and mortality for individuals with DM, emphasis on treatment strategies aimed at reducing cardiac complications secondary to DM are essential for improving the poor outcome of these patients. In that regard, the recent emergence of evidence demonstrating the efficacy of antioxidants as prophylactic and therapeutic agents for cardiac complications of DM support a role for antioxidant therapy in protecting against cardiovascular disease inflicted by accentuated oxidative stress intrinsic to the diabetic state. An important consideration regarding antioxidant therapeutic strategies is the measurement of biomarkers of oxidative stress [111]. Implicit in the majority of randomized placebo-controlled clinical trials that have previously explored the benefits of antioxidant supplementation in preventing cardiovascular events is that the antioxidants tested effectively suppressed oxidative stress status but this was never determined [112]. The omission of oxidative stress monitoring and consequent absence of data demonstrating suppression in the levels of oxidative stress in relation to the dose of antioxidants tested in the study participants enrolled in these trials could explain the null results in certain studies. Moreover, evidence of increased oxidative stress was not a criterion for inclusion in these clinical trials; therefore, antioxidants may have benefited only a subset of the participants [111]. The demonstration that antioxidants such as vitamin E and resveratrol do in fact significantly suppress F_2_-isoprostanes and nitrotyrosine (oxidative stress biomarkers) in both humans and animals with increased oxidative stress associated with diabetic cardiovascular diseases may be especially important for the diabetic myocardium, owing to its greater propensity for oxidative stress that leads to the development of HF [51]. In addition, this information could inform future studies evaluating the effects of antioxidants in diabetic cardiac disease states associated with oxidative stress about appropriate doses to evaluate treatment required for a significant effect in this population cohort. Administration of antioxidant supplements beginning early after the diagnosis of DM merits serious consideration as an additional therapeutic modality to be incorporated into the therapeutic armamentarium to reduce the risk of future cardiovascular complications.

## Figures and Tables

**Table 1:** Effect of Vitamin E Supplementation on Left Ventricular Hemodynamic Parameters in Control and Diabetic Groups of Animals

Parameter	Control - Vit E	Control + Vit E	Diabetes - Vit E	Diabetes + Vit E
LVSP (mm Hg)	129.2 ± 0.9	128.2 ± 3.6	99.6 ± 2.3*	111.5 ± 2.0*Ф
LVEDP (mm Hg)	0.6 ± 0.5	0.8 ± 0.7	5.1 ± 0.7*	2.8 ± 0.6Ф
+dP/dt (mm Hg/s)	8379 ± 98	8136 ± 140	5103 ± 241*	5965 ± 254*Ф
-dP/dt (mm Hg/s)	7552 ± 287	8024 ± 224	4071 ± 134*	4700 ± 265*
Heart Rate (bpm)	308 ± 6.0	321 ± 4.6	;208 ± 9.8*	206 ± 4.6*
MAP (mm Hg)	114.7 ± 3.1	115.8 ± 5.1	87.3 ± 2.9*	101.7 ± 3.4*Ф

Values are mean ± SEM of 6-8 animals.

* Significantly different (P<0.05) from control groups with vitamin E (+Vit E) and without vitamin E (-Vit E) supplementation.

Ф Significantly different (P<0.05) from the un-supplemented diabetic group. Reprinted from Ref. 53 (Copyright 2007), with permission from Elsevier.
